# A Case Report on 46,XX Male Difference of Sex Development

**DOI:** 10.7759/cureus.68741

**Published:** 2024-09-05

**Authors:** Guilherme Vaz de Assunção, Beatriz T Silva, Miguel Saraiva, Rui Carvalho

**Affiliations:** 1 Department of Endocrinology, Diabetes and Metabolism, Centro Hospitalar Universitário de Santo António, Unidade Local de Saúde de Santo Antônio de Santo António, Porto, PRT

**Keywords:** 46 xx male syndrome, 46 xx testicular dsd, bilateral gynecomastia, differences of sexual development, erectile dysfunction, karyotype test, loss of libido

## Abstract

The 46,XX male syndrome is a very rare disorder/difference of sex development (DSD). Characterized by a 46,XX karyotype with a male phenotype and various abnormalities, including virilized external genitalia, small testes, hypergonadotropic hypogonadism, and azoospermia. Primarily described in small population studies and clinical reports, much remains to be understood about the prevalence of clinical manifestations, treatment outcomes, and long-term follow-up in this disorder. Here we describe a 24-year-old male who sought medical attention due to a history of erectile dysfunction, associated with a loss of libido, impaired concentration, difficulty sleeping, and bilateral gynecomastia. He and his family had no relevant medical history. On physical examination, the patient had a normal development of secondary sexual characteristics but presented with bilateral testicular atrophy with a volume of 6 ml per testis. A testicular and abdominal ultrasound were performed confirming testicular atrophy and finding no other abnormalities. Laboratory analysis revealed a hypergonadotropic hypogonadism with normal prolactin, thyroid stimulating hormone, hemoglobin, hematocrit, and kidney and liver function. The spermiogram, performed twice, revealed azoospermia. A bone densitometry was also performed, reporting osteopenia in the lumbar spine and left hip. A karyotype test was performed revealing a 46,XX (*SRY*-positive) DSD. The patient started on therapeutic supplementation with testosterone showing marked improvement of his libido, erectile dysfunction, and return of testosterone to levels within range. The patient and his partner were referred to infertility outpatient care and subsequently opted for in vitro fertilization using a sperm donor. This case report highlights the need for clinical practical awareness of this rare disorder and its wide phenotypical spectrum while also focusing on important aspects of the current literature regarding its approach and treatment. The limited data on long-term management suggest that there is a need for specialized multicenter follow-up not only to ensure a better understanding of this disorder but also to provide a better care on the quality of life and healthy well-being of this patients.

## Introduction

Disorders/differences of sexual development (DSD) is a general term used to describe a group of rare congenital conditions characterized by anomalous chromosomal, gonadal, and genital sex development [1]. Although chromosomal sexual determined, the 46,XX DSD encompasses a group of conditions resulting from abnormal gonadal development or irregular androgenic hormonal secretion. The most prevalent is congenital adrenal hyperplasia (CAH) with its many types of enzyme deficiencies, but there are even rarer conditions [2]. One such case is the 46,XX male syndrome or 46,XX testicular DSD. This rare condition has a prevalence of one in 20,000 [1-3].

Its primary pathogenetic hypothesis involves the translocation between the Y and X chromosomes during paternal meiosis, resulting in the imprinting of the *SRY *gene onto the X chromosome, promoting fetal and postnatal exposure to elevated amounts of androgens [1,3]. The individuals affected by this disorder can exhibit a range of phenotypic characteristics predominantly characterized by the presence of virilized male external genitalia but with reduced testicular volume, hypergonadotropic hypogonadism, and azoospermia. The primary reason these patients seek medical attention is infertility issues [1,3].

Owing to its rarity, complexity, and the typical timing of diagnosis, this disorder presents a challenge to any clinician. We present a new case of 46,XX male DSD and provide an insightful analysis of the current literature on the pathogenetic mechanisms, clinical presentation, treatment approaches, and long-term follow-up on this DSD subgroup.

## Case presentation

A 24-year-old male was referred to our medical outpatient care, primarily complaining of a history of erectile dysfunction for approximately one year. He also reported a loss of libido, bilateral gynecomastia, mild asthenia, loss of interest or pleasure in daily activities, anxiety, impaired concentration, and difficulty sleeping. His medical history did not reveal any significant illnesses. The patient had no history of cryptorchidism, testicular trauma, or infections such as parotiditis. His job at the time had no hazardous dangers such as prolonged contact with radiation sources or managing cytotoxic agents. He also denied taking any chronic medication. His family had no known history of genetic or endocrine pathologies.

On physical examination, he had a weight of 69 kg and height of 162 cm, with a body mass index of 26.3 kg/m². His blood pressure, cardiopulmonary auscultation, and abdominal palpation were normal. The patient presented a normal development of his secondary sexual characteristics, including a normal distribution and density of body hair and penile size (flaccid penile length of 7.9 cm), deep voice, and, as mentioned above, he had bilateral gynecomastia, confirmed with a breast ultrasound. Observation of the genitalia revealed bilateral testicular atrophy with a volume of 6 ml per testis with no other alteration. This was confirmed by a testicular ultrasound, finding no other abnormalities including obstructive azoospermia. An abdominal ultrasound was also performed, finding no signs of alterations.

Laboratory analysis revealed an hypergonadotropic hypogonadism with a luteinizing hormone (LH) of 11.04 mIU/mL (reference range (RR) 1.3-12.9), a follicle-stimulating hormone (FSH) of 30.81 mIU/mL (RR 0.9-15.0), a total testosterone (TT) of 1.56 ng/mL (RR 2.7-10.7) and a free testosterone (FT) of 7.6 pg/mL (RR 8.8 - 27.0). He also had a normal prolactin and thyroid-stimulating hormone (TSH). Hemoglobin and hematocrit were 14.5 g/dL and 42%, respectively, fasting glycemia was 78 mg/dL and total cholesterol was 199 mg/dL with normal hepatic and kidney function (Table 1). All of the mentioned laboratory parameters were confirmed with a second evaluation. The spermiogram, performed within seven days of abstinence, revealed a normal volume of semen (2.8 mL) and azoospermia. This was confirmed after a second sample. A bone densitometry was also performed, reporting osteopenia in the lumbar spine and left hip.

A karyotype test was performed revealing the following alteration: 46,XX. ish ins(X,Y)(p2;q11.2)(*SRY*+). Fluorescent in situ hybridization (FISH) was carried out using an *SRY* probe revealing the sex-determining region Y (*SRY*) on the short arm of the X chromosome. Thus, making the diagnosis of a 46,XX (*SRY*-positive) DSD. A testicular biopsy was proposed to the patient but he refused.

The patient was started on a mixture of testosterone esters (Sustenon®), reaching the stable doses of 250 mg every four weeks, presenting meaningful improvement of his libido, erectile dysfunction, and remaining complaints and the return of serum testosterone to levels within the normal range (Table 1). He also had an improvement in his bone mineral density and hasn’t had any relevant clinical problems since the beginning of the treatment. The patient and his partner were referred to infertility outpatient care and subsequently opted for in vitro fertilization using a sperm donor.

**Table 1:** Laboratory results at the initial appointment and following the initiation of supplementation *Blood sample for analysis was collected midway through the testosterone administration cycle ALP: alkaline phosphatase; AST: aspartate aminotransferase; ALP: alkaline phosphatase; ALT: alanine aminotransferase; FSH: follicle-stimulating hormone; GGT: gamma-glutamyl transferase; HDL: high-density lipoprotein; LDL: low-density lipoprotein; LH: luteinizing hormone; TG: triglycerides; TC: total cholesterol; TT: total testosterone; FT: free testosterone; TSH: thyroid-stimulating hormone; PRL: prolactin; PSA: prostate-specific antigen;

Laboratory analysis of blood sample (s)	At Presentation	Shortly after starting supplementation*	Reference range
FSH	30.81	0.48	0.9 – 15 mIU/mL
LH	11.04	0.06	1.3 – 12.9 pg/mL
TT	1.56	4.55	2.7 – 10.7 ng/mL
FT	7.60	11.3	8.8 – 27.0 pg/mL
TSH	1.42	1.25	0.27 – 4.2 µIU/mL
Estradiol (E2)	37.5		10 – 56 pg/mL
PRL	11.2		4.04 – 15.2 ng/mL
Haemoglobin	14.5	15.0	13.0 – 17.0 g/dL
Haematocrit	42.0	42.8	40-50%
Fasting Plasma Glucose	78	77	mg/dL
Total Cholesterol	199	208	mg/dL
TG	54	31	mg/dL
HDL cholesterol	53	52	mg/dL
LDL cholesterol	137	139	mg/dL
PSA	0.146		< 4.0 ng/mL
Creatinine	0.7		0.7 – 1.2 mg/dL
Urea	40		10 – 50 mg/dL
AST	22		10 – 34 IU/L
ALT	33		10 – 44 IU/L
GGT	20		10 – 66 IU/L
AFL	82		45 – 122 IU/L

## Discussion

The 46,XX male syndrome is a very rare DSD typically diagnosed in adulthood during infertility consultations [1,4,5]. The diagnosis of this syndrome is based on clinical findings, that can have a considered spectrum of phenotypical presentations, hormonal analysis, and cytogenetic tests [1].

The pathological features of this disorder depend largely on the presence of the *SRY*. This gene is widely considered a master regulator acting as a repressor of the female pathway and promoting the development of testes and the differentiation of the Sertoli cells [1,2,6]. The accepted pathogenetic cause is hypothesized to be the translocation of Y to X chromosome during meiosis, or even more rarely to an autosome, granting the 46,XX individuals *SRY* positive status, prevalent in approximately 80-90% [1,2]. On the other hand, there are *SRY-*negative patients. It is now acknowledged that many other genes have an important role in gonadal differentiation: *SOX9*, *SOX3*, *DAX1*, *NR5A1, *or *WNT4,* just to name a few examples [2,6]. It has been suggested that mutations in these genes located downstream of *SRY* in the sex-determining pathway may contribute to the development of *SRY*-negative patients [5]. The 46,XX male difference is probably the result of multiple determinants on the genes cascade that may increase the expression of genetic pathways implicated in the testicular differentiation and/ or a lack of expression of genetic pathways involved in ovarian differentiation [2,6].

As outlined above, there is a wide spectrum of phenotypically male characteristics, although the most frequent, due to the high prevalence of *SRY*-positive individuals, are the usual male phenotypes with normal development of secondary sexual characteristics, bilateral testicular atrophy, hypergonadotropic hypogonadism and azoospermia [4]. These features make the diagnosis before puberty extremely difficult, unlike the *SRY*-negative patients, due to the higher prevalence of genitalia abnormalities, making the clinician suspect of a DSD at a much precocious age [1].

Unlike our patient, the majority of the cases that seek medical help and are subsequently diagnosed, are the ones that complain about infertility or poor testicle development [1,4-6]. It is estimated that this disorder accounts for 2% of male infertility cases [7]. In a cohort of 144 adult patients with 46,XX male DSD, 100% had azoospermia [8]. In another study, when compared with fertile men, all 46,XX males had azoospermia and smaller median semen volume [9]. The findings of immature testes with germinal aplasia, Leydig and Sertoli cell hyperplasia and lack of spermatogonia or spermatozoa render the reported conclusion of azoospermia for all 46,XX male difference patients [6].

Loss of libido, apathy, and erectile dysfunction were the main clinical manifestations that prompted our patient to seek medical advice. Some cohort studies and a systematic review on young adults with 46,XX male DSD, reported that 90% had normal erectile function and 100% had preserved libido [1,4].

On physical evaluation, the majority of patients have no apparent alterations with virilized external genitalia, although hypospadias and cryptorchidism can occur at a young age, ranging from 4.1% to 10% and 9.7% to 15% of the individuals, respectively [1,2,8]. Upon initial physical observation, the only findings in our patient were gynecomastia and bilateral atrophic testes. It is important to mention the relevance of the testicular inspection and palpation since in the cohort by Chen et al., almost 100% had bilateral atrophic testes with less than 15 ml on both sides [8]. It has been speculated that during puberty, the absence of the Y chromosome and genetic pathways needed for testicular maintenance may lead to progressive germ cell loss, azoospermia, and consequently reduced testicular volume. As a result, testosterone levels might be normal during adolescence, but decrease in adulthood [2]. Of note, and relative to our patient, there are some reports on lower than average height in the 46,XX male DSD population when compared to the general population (height of 171 cm according to the World Health Organization growth reference standards) [5], with the presumed cause being the lack of expression of specific growth hormone receptors genes in the Y chromosome. This is yet to be confirmed [1,6].

Laboratory testing usually finds a hypergonadotropic hypogonadism, normally with high FSH and high LH, a low inhibin B, and low serum testosterone [2,3]. Although our patient did not present with a high LH, there have been some literature reporting the same results [8]. An abdominal and testicular ultrasound can be useful to rule out any other congenital abnormalities in this area and assess the presence or absence of Müllerian ducts [1].

The definitive diagnosis is made by karyotype and FISH testing [1,3], and differential diagnosis should be performed between 46,XX ovotesticular DSD (coexistence of functional ovarian and testicular tissue in the same individual) and testicular DSD. This is made through a testicular biopsy and histological analysis, although, 90% of the individuals with ovotesticular DSD will be *SRY *negative, with a high prevalence of diagnosis in the neonatal period, displaying an immensely variable phenotypic presentation depending on the histological findings [2,4,10].

Testosterone replacement therapy is widely used in hypogonadal men to provide a sense of well-being, better sexual desire and performance, increase lean body mass and muscular strength, and also improve bone mineral density. Before starting this treatment, clinicians should access the prior medical history of prostate or breast cancer, decompensated heart failure, prior stroke or acute myocardial infarction, sleep apnoea, thrombophilia, or a high hematocrit [11]. Surveillance during treatment with testosterone presumes a regular assessment of the new onset of hypogonadism-related signs and symptoms. Besides measuring serum testosterone levels, clinicians should also consider assessing hematocrit, serum prostate-specific antigen, lipid profile, and liver function [3].

On the long-term follow-up of these individuals, data is very scarce, particularly on mental health. The rarity of this group and their frequent loss of follow-up are the main reasons for this issue [2,4].

On their quality of life, little is known. The dsd-LIFE study that assessed the quality of life in DSD patients had a cohort of 1,040 individuals, 27 of them with non-CAH 46,XX DSD, and only 2.4% were 46,XX male. Nonetheless, when compared to a healthy reference population the results showed a lower physical and psychological health [4,12].

Also, the incidence of gender dysphoria and gender transition remains poorly demonstrated in this group, although some small studies showed a prevalence of 11-15% for gender dysphoria in the 46,XX DSD population [4].

In the dsd-LIFE cohort, 90% of the individuals reported good health, compared to the 46,XX DSD group, where 16.7% reported poor health [12]. In the same study, cardiovascular and metabolic outcomes were also measured, comparing DSD individuals with a group control. It demonstrated an increased cardiovascular risk with 15% of the cohort having at least one cardiovascular disease. As for the 46,XX male, there were no signs of increased cardiovascular risk but, as noted above, the small number of non-CAH in the study should be taken into consideration [12].

At the time of diagnosis, our patient presented with osteopenia of the hip and lumbar spine with later improvement in both areas. This result is consistent with the literature which shows an improvement of almost 50% in bone mineral density after initiation of hormone replacement therapy [4].

Another non-communicable disease with limited data in this population is the cancer prevalence. In DSD, the presence of the Y chromosome is a risk factor for gonadal tumors, especially germ cell tumors, being very rare in the 46,XX male DSD patients [2,10,13,14]. In this population, there have been very few reports of germ cell tumors, dysgerminomas, gonadoblastomas, and non-germ cell tumors [4,6,10,13-15]. In the 46,XX DSD subgroup, ovotesticular gonadal dysgenesis has a higher risk associated with the incidence of germ cell tumors [2,3,10,13]. It has been hypothesized that some genes such as the *WT1* and the gene encoding the protein TSPI could have a role in the development of gonadal tumors [3,7], but the degree of testicular dysplasia could also play an important part in this risk [6].

In 46,XY testicular dysgenesis, orchiectomy is recommended before puberty, due to the high risk of gonadoblastoma [6]. In the ovotesticular disorder, since in most cases there is no prediction on the future gender, gonadectomy should not be performed until pubertal age [2]. The aesthetic aspect may also influence the decision to undergo orchiectomy since nearly all patients with 46,XX male difference have bilateral testicular atrophy. In such cases, replacement with bilateral testicular prostheses could be helpful for psychological well-being. Overall, although data after puberty is scarce, the risk of gonadal tumors in non-CAH 46,XX DSD appears to be low, and prophylactic gonadectomy is not recommended in this subgroup [2,4,10].

Other parameters lacking information are the mortality and morbidity rates. In a study including 44 men with 46,XX DSD, each individual was compared with a randomly selected age-matched control group. The results showed no differences in mortality and, after the exclusion of patients with congenital malformations and endocrine diseases, no differences in morbidity [15]. Unfortunately, as with many clinical issues mentioned, the number of individuals on follow-up were limited and more data is needed to provide effective conclusions.

Fertility options are one of the more important subjects in the follow-up of 46,XX Male DSD since these individuals lack the *AZF* loci on the long arm of the Y chromosome [3]. One of the main responsibilities of a clinician is to explain the potential fertility options. As mentioned above, it is well reported in the literature that 46,XX male disorder has a very high prevalence of azoospermia [1,3,4,8]. In the dsd-LIFE cohort, most of the patients with 46,XX male disorder were satisfied with the discussion they had with their clinician about fertility treatment. Of the six individuals, two of them had children by the only possible means in these cases: one by adoption and the other by sperm donation through assisted reproductive technology [6,12]. Our patient was referred to infertility outpatient care and, alongside his partner, decided to conceive through sperm donation. Until this day he showed contentment with all of the steps of the process.

## Conclusions

The presented case report provides insight into a new clinical case of 46,XX male DSD, where the primary reason for referral was not infertility but erectile dysfunction and other symptoms associated with hypogonadism while comparing additional findings with the ones reported in the literature. It’s important to perform a thorough medical history, assess physical alterations, and address the predominant and uncommon manifestations to provide a better understanding of the phenotypical spectrum of this DSD. In this regard, clinicians have a golden opportunity to offer the best medical approach as soon as they establish contact with the patient.

Despite our patient not presenting any complaints or relevant comorbidities since the initiation of testosterone supplementation, we also addressed the long-term follow-up, concluding that the literature contains limited significant data, regarding quality of life, morbidity, and mortality in this population. A continuous follow-up in a specialized center with a multidisciplinary team could solve this issue, offering the possibility of pediatric transition to adult care and management of chronic treatment while gathering much-needed knowledge on non-communicable diseases and their evolution with aging in this DSD subgroup. We also cannot disregard the psychological impact of a DSD diagnosis. Continuous counseling support can be beneficial. For individuals with 46,XX male DSD, such support is especially valuable when discussing fertility options, as it can ensure a better outcome in their quality of life and healthy well-being.
